# Super-Resolution Microscopy: A Technique to Revolutionize Research and Diagnosis of Glomerulopathies

**DOI:** 10.1159/000528713

**Published:** 2022-12-18

**Authors:** Florian Siegerist, Vedran Drenic, Thor-Magnus Koppe, Nihal Telli, Nicole Endlich

**Affiliations:** ^a^Institute for Anatomy and Cell Biology, University Medicine Greifswald, Greifswald, Germany; ^b^NIPOKA GmbH, Greifswald, Germany

**Keywords:** Super-resolution microscopy, Fluorescence microscopy, Quantitative image analysis, Glomerular disease

## Abstract

**Background:**

For decades, knowledge about glomerular (patho)physiology has been tightly linked with advances in microscopic imaging technology. For example, the invention of electron microscopy was required to hypothesize about the mode of glomerular filtration barrier function.

**Summary:**

Super-resolution techniques, defined as fluorescence microscopy approaches that surpass the optical resolution limit of around 200 nm, have been made available to the scientific community. Several of these different techniques are currently in use in glomerular research. Using three-dimensional structured illumination microscopy, the exact morphology of the podocyte filtration slit can be morphometrically analyzed and quantitatively compared across samples originating from animal models or human biopsies.

**Key Messages:**

Several quantitative image analysis approaches and their potential influence on glomerular research and diagnostics are discussed. By improving not only optical resolution but also information content and turnaround time, super-resolution microscopy has the potential to expand the diagnosis of glomerular disease. Soon, these approaches could be introduced into glomerular disease diagnosis.

## Introduction

The first written description of glomeruli can be found in 1666 in “De Viscerum Structura Exercitatio Anatomica” by Italian anatomist Marcello Malpighi, who used an early version of the simple light microscope invented by Robert Hook to investigate multiple organs and organisms. Malpighi described the glomerulus as a small, dark corpuscular structure on the kidney surface [1]. However, the invention of modern light microscopy in the 19th century was necessary for Sir William Bowman to further describe the glomerular tuft and especially the cells surrounding the glomerular tuft in his work “On the structure and use of the Malpighian bodies of the kidney” in 1842 [2]. He extensively described these structures that later were named “Bowman's capsule.” In addition, Bowman was the first to coin the term “basement membrane” for a structure that was described by him in the kidney and other organs as an “extremely thin, transparent, and homogeneous lamina, simple and entire, without any (…) appearance of structure” [2]. Seeing these early examples, it is not surprising that developments in microscopic technology and knowledge in the field of glomerular biology have been tightly linked for decades. As an example, the invention of electron microscopy, a technique that was able to resolve the glomerular filtration barrier in great detail, was needed to hypothesize about the detailed action of glomerular filtration. Since the 1960s, Karnovsky's seminal work on glomerular ultrastructure has acted as a major driver for glomerular research. His group described the filtration slit as an isoporous structure between podocyte foot processes [3, 4]. Using freeze-fractured scanning electron microscopy, the group described regularly patterned bridges spanning across the filtration slit which later were found to be the ultrastructural correlate of nephrin homodimers spanning the filtration slit [5]. With the discovery that proteins encoded by genes associated with proteinuria were localized to the filtration slit diaphragm of the slit diaphragm result in proteinuria, the importance of this intricate cell-cell contact for the maintenance of the glomerular filtration barrier has been underlined [6, 7]. Irrespective of this, it has been known for decades that many structural alterations of the glomerulus lead to functional changes. Especially for podocytes, even slight changes in the three-dimensional (3D) architecture will lead to impairments in the selective permeability of the kidney filter [8]. These stereotypical changes are either a reaction to mechanical challenges induced by glomerular hypertension or hyperfiltration or can be the result of cellular injury and podocyte depletion. Therefore, considerable efforts have been put into morphometrically analyzing changes in this structure.

## Expanding the Microscopy Imaging Toolbox

**Fig. 1:**
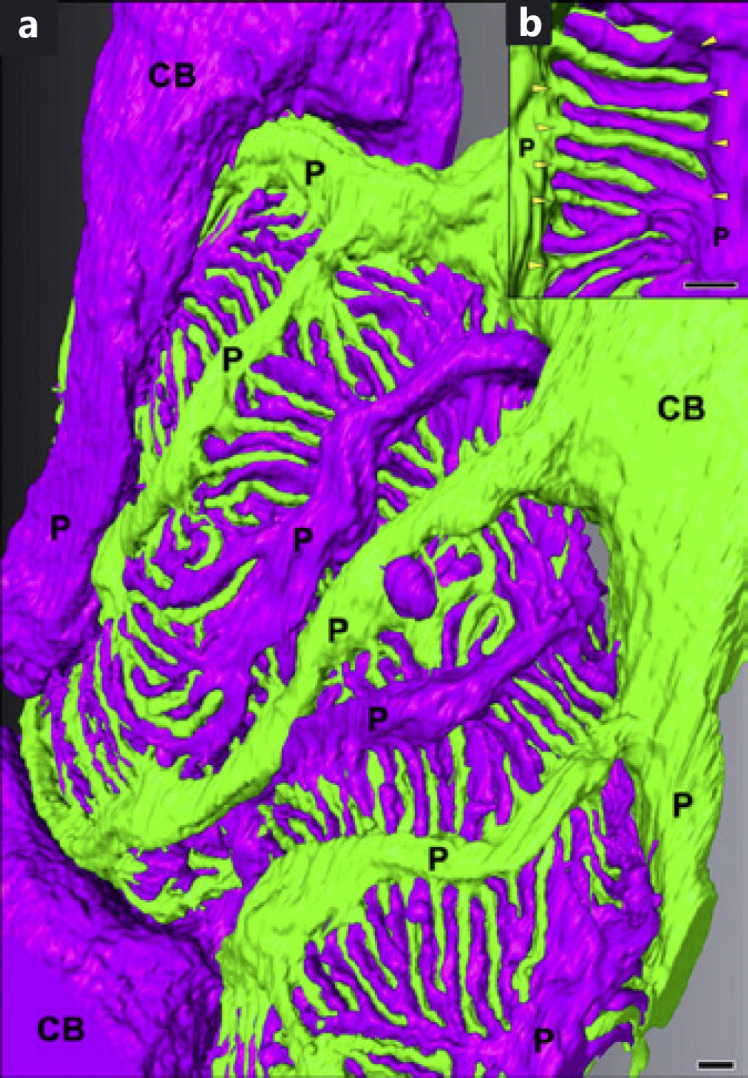
Volumetric rendering of rat podocytes from a serial block-face electron microscopy dataset. Image **(a**) shows three neighboring podocyte cell bodies (CB) that give rise to multiple primary processes (P, major processes). Single interdigitating foot processes with their base at the major processes (arrowheads in (**b**)) are shown. This figure has been adapted from Ichimura et al. [12], which is published under a Creative Commons 4.0 license https://creativecommons.org/licenses/by/4.0/.

To date, in the pathohistological analysis of kidney biopsies, transmission electron microscopy (TEM) is still an important tool [9]. In routine diagnostics, TEM is used to evaluate the localization and morphology of deposits, as well as the general appearance with features such as the electron density or thickness of the glomerular basement membrane (GBM). Additionally, podocyte foot process effacement is evaluated with TEM. While TEM provides the highest resolution, it has drawbacks in terms of throughput and limitations in morphometric analysis. Typically, <5 glomeruli will be evaluated by TEM. Findings from such limited material may lack generalizability to the entire biopsy or the whole kidney of the respective patient. Additionally, due to physical sectioning, a geometric bias is introduced, meaning that the highly complex structures like podocyte foot processes will be sectioned in different orientations, therefore making it hard to measure the exact widths of the foot processes. This issue makes morphometric analysis of 2D single sections very challenging [10]. Due to these issues, the description of the degree of foot process effacement is typically either semi-quantitative or even binary [11]. To overcome this, several researchers have applied serial sectioning 3D electron microscopy to reconstruct entire interdigitating podocytes. Using very elegant 3D reconstructions of serially acquired electron microscopy, Ichimura et al. [12, 13] and others [14, 15] reconstructed the foot process structure of entire interdigitating podocytes (Fig. 1a, b). With this approach, Ichimura and colleagues newly described ridges linking major processes to the GBM, which themselves form contacts with adjacent foot processes. Unfortunately, because of prolonged imaging sessions and even more time required for the manual segmentation of the image stacks, this technique plays an important role for basic research purposes, but it is unlikely that this enormous effort can be transferred into translational research or even routine diagnostic kidney biopsy evaluation.

As healthy human podocyte foot processes have a diameter under 250 nm, for decades, their analysis has been an exclusive task for electron microscopy. Nowadays, several fluorescence microscopy techniques are available that surpass the optical resolution limit. These techniques are summarized under the term super-resolution microscopy (SRM). While this term is only descriptive of the general ability to break the diffraction limit, it summarizes a variety of entirely different technical approaches to achieve this. A comprehensive overview of the multitude of SRM techniques is beyond the scope of this article and can be found elsewhere [16]. In glomerular research, the most recognized techniques are 3D-structured illumination microscopy, stimulated emission depletion (STED) microscopy, single-molecule localization microscopy (SMLM), and expansion microscopy (ExM). These individual techniques are further discussed below.

## Three-Dimensional Structured Illumination Microscopy

**Fig. 2:**
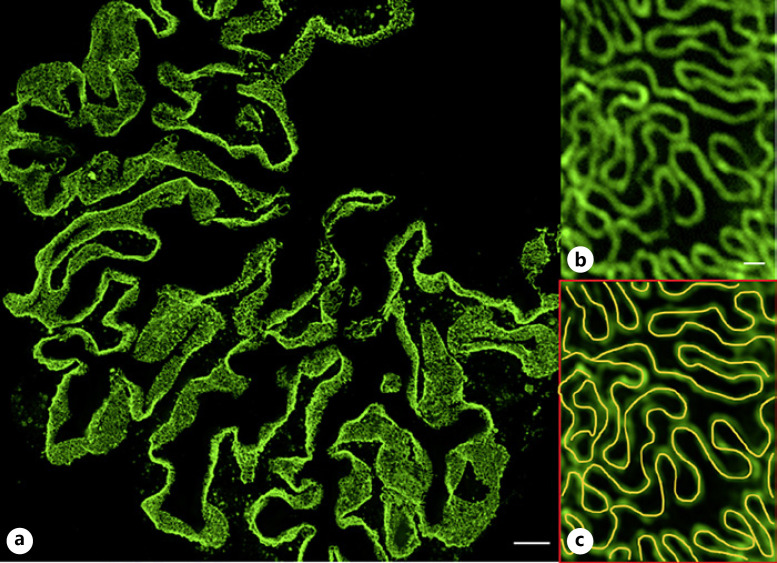
PEMP principle: a super-resolved image of an entire glomerular cross-section stained with an antibody against a filtration slit component (here nephrin) is acquired (**a**). **b** Single podocyte foot processes can be reliably distinguished. The PEMP algorithm detects the lines of the filtration slit (yellow in (**c**)) and measures the total length of the filtration slit relative to the total area of the glomerular capillaries. This value is described as the filtration slit density (FSD). The scale bar represents 10 μm in the overview and 200 nm in the magnification.

In three-dimensional structured illumination microscopy (3D-SIM), a sample is illuminated with a patterned laser beam. Interference of the illumination pattern with the biological specimen will create so-called Moiré patterns that have a lower frequency than both the illumination pattern and the specimen. These low-frequency patterns can be identified with a classic wide-field detection system. For every z-plane, 15–25 single images with different orientations of the illumination pattern are acquired, and the super-resolved image is then digitally reconstructed. A typical 3D-SIM system achieves around 120 nm lateral resolution on tissue sections which is enough to resolve the intricate podocyte foot processes and their respective changes in disease. In 2016, Pullman and colleagues used 3D-SIM to resolve individual podocyte foot processes stained with podocin in human fresh frozen and cryosectioned kidney biopsies and showed differences like linearization of the filtration slit in nephrotic syndrome samples [17]. Shortly after, we published a similar approach, which was optimized for routinely generated formalin-fixed and paraffin-embedded (FFPE) material with anti-nephrin immunofluorescence. To automatically analyze the resulting data, we established an automated morphometric approach to quantitatively analyze the degree of foot process effacement in these samples. To do so, an algorithm was established that determines the filtration slit density (FSD) - meaning the length of the filtration slit per glomerular capillary surface (Fig. 2a-c). As we have shown, this approach, later named podocyte exact morphology measurement procedure (PEMP), is less biased and highly correlated with the established marker of foot process width [10].

In follow-up work, we optimized the technique to also generate FSD data from rodent animal models which is challenging as the foot process width is significantly lower than that of humans [18]. Herein, we could demonstrate significant changes in the filtration slit morphology in sub-proteinuric palladin knockout mice in comparison to wild-type littermates. One of the greatest benefits of 3D-SIM is its near out-of-the-box compatibility with existing workflows. One important thing to consider is the use of high-quality materials like high-precision coverslips and bright, bleach-stable fluorophores [19]. An additional benefit of 3D-SIM is the ability to easily multiplex multiple targets on one slide to correlate local changes of the filtration slit morphology with the abundance of additional marker proteins. We have used this approach to evaluate the evolution of foot process effacement and the conserved dedifferentiation of the filtration slit toward a tight junction during disease development. We showed that in the early phase, the tight junction protein claudin-5 is recruited to the filtration slit and could therefore serve as an early marker that predicts podocyte foot process effacement [20].

**Fig. 3:**
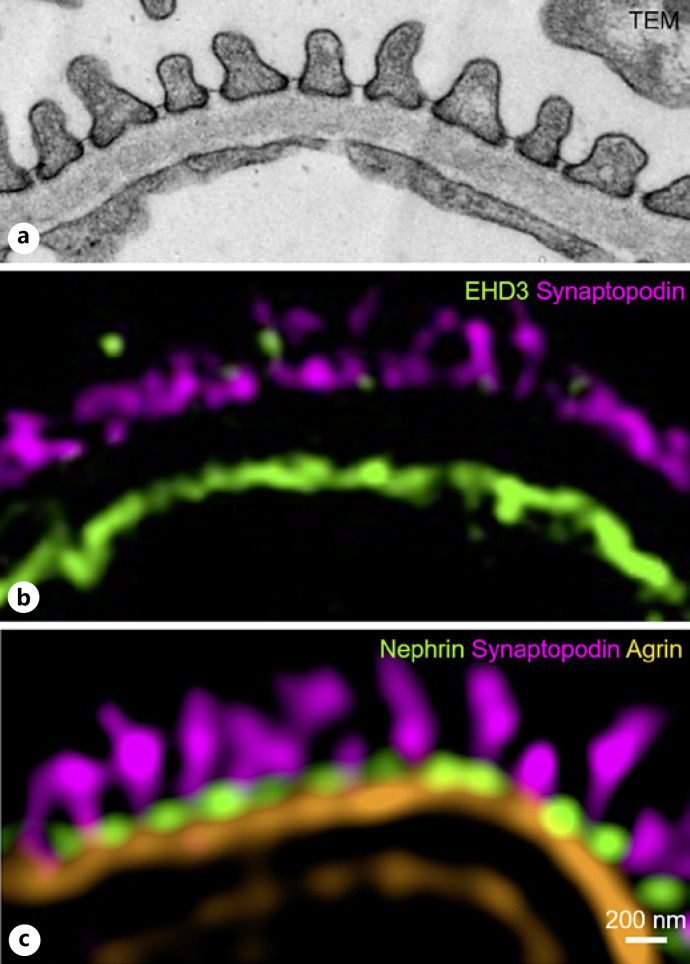
**a**−**c** Multiplexed 3D-SIM reveals morphometric features of the glomerular filtration barrier similar to TEM. Using multiplex immunofluorescence staining for podocytes (synaptopodin, nephrin), endothelial cells (EHD3), or markers for components of the GBM (agrin), its thickness can be structurally analyzed, similar to TEM. Additionally, these findings can directly be correlated with the respective FSD values.

**Fig. 4:**
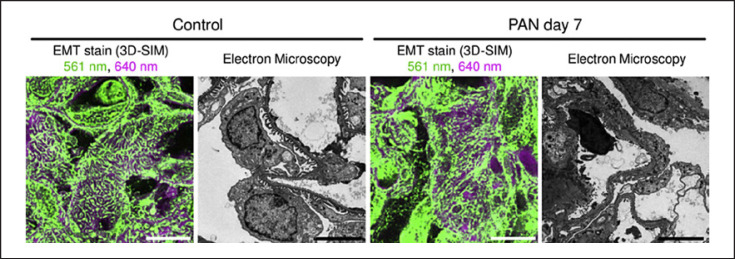
3D-SIM of Elastica-Masson trichrome-stained rat kidney sections compared with TEM. While healthy interdigitating foot processes can be distinguished in the 3D-SIM images of control animals, broad destruction of foot process morphology can be seen 7 days after PAN injection. This figure has been adapted from Matsumoto et al. [28], which was published under a Creative Commons 4.0 license https://creativecommons.org/licenses/by/4.0/.

In addition to podocyte foot process morphology, 3D-SIM can retrieve additional information that is a typical domain of TEM (Fig. 3a). With a simple combination of markers for endothelial cells (EHD3, Fig. 3b) or the GBM (agrin, Fig. 3c, unpublished 3D-SIM data of a murine kidney section by our group), the width of the GBM can be easily evaluated in parallel to PEMP analysis. To date, PEMP has emerged as an important tool and has been used in several different publications to determine the FSD in animal models [18, 20, 21, 22, 23, 24, 25], human samples [26], or even organoids [27]. Additional interesting development was presented by Matsumoto and colleagues, who imaged stained kidney sections with regular histological dyes periodic acid Schiff (PAS) and Elastica-Masson trichrome on FFPE material. Using the differential affinity of these dyes and their respective fluorescence spectra, several morphometric features could be retrieved from FFPE material: podocyte foot processes were visualized from Elastica-Masson trichrome staining, and the GBM integrity was investigated in PAS staining [28] (Fig. 4). This method is fast and also allows for direct integration of standard bright-field and SRM and in principle could be possibly used for morphometric assessment with PEMP. For quantitative comparison, the group used Fourier transformations to retrieve the frequency information which in a periodic structure like podocyte foot processes changes upon foot process effacement. However, correlations with established markers like foot process width or FSD are required for a definite conclusion on this approach.

## STED Microscopy

Put simply, the STED principle relies on forced fluorescence depletion in a defined area around the point of interest. A donut-shaped laser beam is used to deplete the fluorescence emission of a second central laser beam. Therefore, the size of the point of fluorescence emission is directly related to the power of the depleting laser. This directly leads to limitations of this technique; the gain of resolution highly relies on how well the “depletion donut” can be illuminated into the sample. As inhomogeneities of the optical refractive index within the tissue will lead to distortion of the STED illumination, the resolution will not be optimal in the entire volume of these standard-processed samples. This is one of the reasons why STED microscopy is error prone with routinely generated FFPE kidney material, which at the moment hinders application within routine workflows [29]. To overcome this issue, in 2016, Unnersjö-Jess and colleagues deployed hydrogel embedding of fixed tissue in combination with solvent-based optical clearing, which improved imaging conditions significantly. Using this technique, they could clearly resolve the filtration barrier using immunofluorescence staining of the slit diaphragm proteins nephrin and podocin [30].

## Single-Molecule Localization Microscopy

In single-molecule localization microscopy (SMLM), fluorophore excitation is not separated spatially but temporally. In the glomerular field, the most frequently used approach is direct stochastic optical reconstruction microscopy. Herein, fluorophores are illuminated with high laser power in oxygen-free reducing conditions. Dependent on the laser energy, several fluorophores can cyclically alternate between photon emission (ON state) and darkness (OFF state). When continuously imaged, rapid switching between these states can be imaged as blinking. As these blinking events are stochastically distributed across the sample, the acquisition of multiple thousand frames contains single-molecule localization information. When, in such a dataset, blinking events are so sparse that one blinking event corresponds to one fluorophore, the center of the localization of that fluorophore can be approximated. Therefore, the localization precision directly depends on the number of photons emitted. Typically, SMLM is a domain for rather simplified biological samples like cultured cells or even, as we have shown, purified protein or immobilized viral particles [31, 32].

While SMLM offers the highest resolution among these abovementioned super-resolution techniques, it is also a rather advanced technique with a large number of critical steps involved. This corresponds to a rather small number of publications in which SMLM has been successfully used in the field since it has been introduced to the glomerular disease research community by Suleiman and colleagues in 2013 [33, 34].

Additionally, with the application of correlative super-resolution and platinum-replica scanning electron microscopy, they combine the best of two worlds [35]. In 2021, Garcia et al. [36] showed a promising approach using SMLM to resolve IgG deposits in the GBM in routinely prepared biopsies of patients suffering from membranous nephropathy. In this work, the researchers demonstrate the possibilities of this powerful tool in routine histopathologic diagnosis [36].

## Expansion Microscopy

**Fig. 5:**
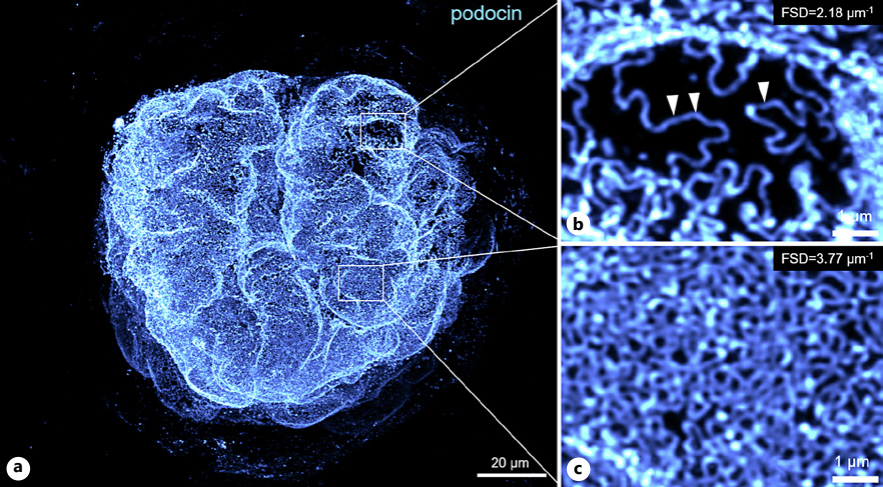
Tissue clearing and expansion enables super-resolved analysis of the filtration barrier using confocal laser scanning microscopy. Image (**a**) shows a z-projection of a 35 μm volume of a mouse glomerulus. Primary anti-podocin antibodies were detected using Alexa Fluor 488-conjugated nanobodies. **b, c** Magnifications of two areas of the same glomerulus are shown. While magnification in (**b**) shows an area with broad podocyte foot processes as shown by more linearized podocin-stained filtration slits (arrowheads) and a rather low filtration slit density (FSD, 2.18 μm^−1^), the magnified area in (**c**) shows normal appearance of podocyte foot processes with a higher FSD (3.77 μm^−1^).

While commercially available super-resolution imaging systems are still expensive, which is a hurdle for many potential users of this technique, one approach that aims to overcome this is ExM. Instead of constructing optical systems that resolve smaller structures, ExM works by physically expanding these structures via isovolumetric swelling. Once expanded, existing fluorescence microscopes like wide-field or confocal laser scanning systems can be used to resolve details that are normally not resolvable in native samples. In 2017, Zhao and colleagues applied ExM to FFPE samples from routine diagnostic pathological material with a technique named ExPath. In their study, samples that were processed and stained with H&E were used for expansion [37]. Interestingly, podocyte foot processes could be resolved and were found to be significantly broadened in minimal change disease. Other groups have applied ExM for the visualization of glomerular ultrastructure [29, 38]. While compatible with existing imaging systems, the drawback of this technique is the delicate sample preparation with infusion, polymerization, immunolabelling, and finally swelling of the gels, which do not fit seamlessly into existing pathological workflows. In 2018, Unnersjö-Jess and colleagues combined ExM with STED microscopy to further improve imaging resolution [39]. In 2021, the same group published a surprisingly simple protocol in which fixed kidneys are sectioned with a vibratome, cleared in SDS to dissolve lipids, immunostained, and mounted in a concentrated fructose solution [40]. This induces mild swelling (approximately 1.7-fold) of the samples which leads to enough sample expansion to resolve foot processes. In Figure 5, we show our unpublished data using this protocol. We could visualize intact murine glomeruli of healthy animals (Fig. 5a) with a resolution high enough to resolve both effaced podocyte processes with linearized podocin-stained filtration slits (Fig. 5b) and normal-appearing podocyte foot processes (Fig. 5c) with a standard confocal laser scanning microscope setting. The previously described PEMP approach can also be applied to this method as demonstrated for the two magnified areas in Figures 5b and c. This decreases the dependency on expensive super-resolution systems and opens the field of podocyte foot process morphometry to more groups.

## Artificial Intelligence to Improve Quantitative Image Analysis and Diagnosis

Since its introduction, the spectrum of artificial intelligence has developed several promising applications in the field of quantitative image analysis. During the last few years, histopathologic analysis has become increasingly complex. Therefore, trained neural networks can take part of the increasing workload. Currently, trained networks are primarily used for three different tasks. First, in image segmentation, pixels of an image are classified as either positive or negative for a particular feature. In kidney research, this approach has been used for the virtual microdissection of glomeruli from the tubulointerstitium or segmentation and quantification of fibrotic areas in renal biopsies. Zimmermann and colleagues used this approach to enhance glomerular and podocyte morphometry of immunostained FFPE sections. In their 2021 paper, they showed that morphometric markers such as podocyte depletion allowed previously unknown patient classification, which could provide additional information to determine individual patient risk stratification in the future [41].

Second, in image-to-image translation, a network is trained to predict an image from a given input image. Approaches to predict immunofluorescence staining from transmitted light micrographs of unstained cells have been presented previously [42]. This approach has been applied in PodoSighter, a cloud-based tool that uses a trained network to predict podocyte WT1 or p57 immunofluorescence from PAS sections to then quantify podocyte density in whole-imaging data from rodent models or patient biopsies [43]. In the era of digital pathology, this cloud-based online tool can be a valuable addition to assessing podocyte depletion in routinely prepared whole-slide imaged PAS sections.

Third, a deep learning (DL) network can be used to categorize structures of interest in a scoring system. In a combined hypertensive and diabetic murine kidney disease model, Østergaard and colleagues have nicely shown how a trained network can be used to classify glomerular sclerotic lesions in FFPE sections [44]. One caveat to these promising results is the generalizability of a trained network to new data. If a network is trained on a rather homogeneous dataset, its predictions on heterogeneous real data can be inaccurate. Therefore, care has to be taken to select a well-annotated training dataset that should recapitulate the expected future data as best as possible.

There are different approaches to applying DL to improve SRM. In 2020 and 2021, two independent groups trained DL networks for improved 3D-SIM reconstructions. These networks required fewer raw images for reconstruction and perform well under low-light conditions, which can reduce both photobleaching and imaging time [45, 46]. Using DeepSTORM3D, Nehme and colleagues presented a DL approach to process SMLM data that works with very dense localization data and therefore with a massively decreasing imaging time [47].

For many years, artificial intelligence in quantitative image analysis was a domain of highly specialized computer scientists, and direct application by wet-lab scientists was an issue. This has changed with the efforts of Chamier and colleagues in 2021 [48] to “democratize” DL for microscopy. With ZeroCostDL4Mic, they presented several fully online and easily accessible DL networks for multiple tasks. The networks are embedded in Google Colab notebooks so that the computation is performed in the cloud, therefore, decreasing the hardware and coding demands of the individual scientist.

**Fig. 6:**
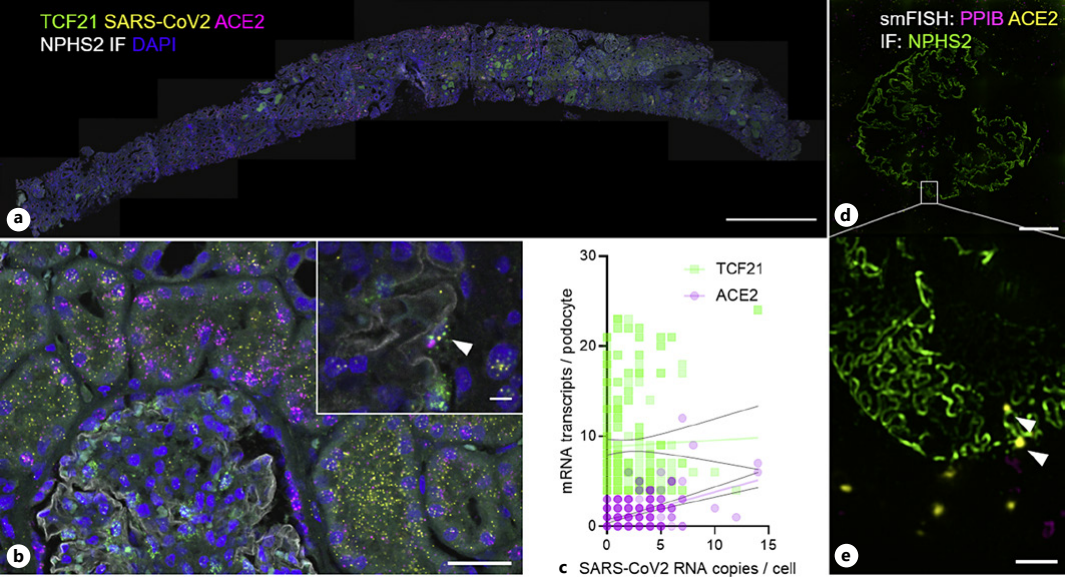
DL enabled single-cell expression analysis. Image (**a**) shows an overview of a longitudinal cross-section of a kidney core biopsy with 3 (m)RNA markers (TCF21 - podocyte marker, SARS-CoV-2 - viral RNA, and ACE2 - entry receptor of SARS-CoV-2) combined with podocin immunofluorescence (NPHS2) and nuclear counterstain. Magnifications are shown in (**b**) which demonstrates the high intracellular abundance of SARS-CoV-2 RNA, mainly in ACE2-positive proximal tubular cells. The insert in (**b**) and the graph in (**c**) show that TCF21-positive podocytes also show intracellular SARS-CoV-2 RNA coexpressed with ACE2. The micrographs in (**d**) with the magnification in (e) show how 3D-SIM can resolve the podocin-stained (NPHS2) filtration slit together with mRNA transcripts. In this area, an injured podocyte with elongated filtration slits can be seen with several ACE2 mRNA spots (arrowheads). The scale bars represent 1 mm in (**a**), 25 μm in (**b**), 5 μm in the magnification in (**b**), 20 μm in (**d**), and 1 μm in (**e**), adapted and reprinted from Siegerist et al. 2022, which was published under a Creative Commons BY 4.0 license https://creativecommons.org/licenses/by/4.0/.

In a first proof-of-principle work, we trained DL networks to segment glomeruli and single cells from kidney biopsies using single glomerular markers and nuclear counterstains, respectively [26]. Subsequently, single-mRNA localizations derived from multiplexed single-mRNA in situ hybridization were annotated for every single cell. We showed that the entire approach is compatible with 3D-SIM, enabling direct integration of single-cell expression analysis with super-resolved single-cell morphometry. This technique now integrates the unbiased analysis of all cells that will be segmented in an entire tissue section. For a typical kidney biopsy section, this is approximately 5,000–10,000 cells. Using this technique, we analyzed SARS-CoV-2 RNA and ACE2 mRNA localization in a patient diagnosed with COVID-19-associated collapsing glomerulopathy (Fig. 6a-b). While the vast majority of viral RNA is localized in ACE2-positive proximal tubule cells, a significant correlation between viral RNA appearance and ACE2 expression could also be found in podocytes (Fig. 6c). Using 3D-SIM, podocytes with elongated podocin-stained filtration slits showed ACE2 expression (arrowheads in Fig. 6d, e), illustrating the combination of SRM and single-cell expression analysis [26].

## Conclusion

SRM has the potential to change the diagnosis of podocytopathies as we know them. It is conceivable that these approaches will soon be developed for application in routine renal biopsy diagnostics. DL methods will further accelerate diagnostic workflows and interpretation, leading to yet unknown sub-categorization of glomerulopathies that will enable high-precision clinical trials or risk stratification for disease progression in individual patients. This will require trials to show clear benefits from these new diagnostic methods in comparison with the current standard diagnostic procedures.

## Conflict of Interest Statement

Florian Siegerist holds shares, Vedran Drenic and Nihal Telli are employees, and Nicole Endlich serves as CEO and holds shares of NIPOKA GmbH, Greifswald, Germany, a startup commercializing PEMP. Thor-Magnus Koppe has no conflicts of interest.

## Funding Sources

This work has been supported by a starting grant of the “Forschungsverbund Molekulare Medizin” and by a scholarship of the “Gerhardt-Domagk Masterclass” of the University Medicine Greifswald to FS. This work has been supported by a grant from the Federal Ministry of Education and Research (BMBF, Grant 01 GM1518B, STOP- FSGS) to NE. This work was generously supported by the Südmeyer fund for kidney and vascular research (“Südmeyer Stiftung für Nieren- und Gefäßforschung”) and the Dr. Gerhard Büchtemann fund, Hamburg, Germany. None of these funding sources had a direct influence on the conceptualization of the article.

## Author Contributions

Florian Siegerist, Vedran Drenic, Thor-Magnus Koppe, and Nihal Telli prepared samples and acquired imaging data shown in Figures 2, 3 and 5, 6. Florian Siegerist and Nicole Endlich conceptualized the review article and wrote the manuscript. All authors approved the final version of the manuscript.
